# Determination of ethyl glucuronide in hair and self-reported alcohol consumption in university students

**DOI:** 10.1007/s12024-023-00727-x

**Published:** 2023-10-05

**Authors:** Jennifer P. Pascali, Arianna Giorgetti, Guido Pelletti, Luca Morini, Susan Mohamed, Marta Barbaresi, Rossana Cecchi, Susi Pelotti, Paolo Fais

**Affiliations:** 1 Department of Medical and Surgical Sciences, Unit of Legal Medicine, University of Bologna, via Irnerio 49, Bologna, Italy; 2 Department of Public Health, Experimental and Forensic Medicine, University of Pavia, Pavia, Italy; 3 Department of Medicine and Surgery, Unit of Legal Medicine, University of Parma, Parma, Italy

**Keywords:** Forensic toxicology, Ethyl glucuronide, Students, Alcohol, Hair, Questionnaire

## Abstract

Young individuals constitute an intriguing population, as their drinking habits are notably shaped by their perception of their peers’ alcohol consumption. Nonetheless, excessive alcohol intake can have detrimental effects on academic performance, interpersonal relationships, and the risk and severity of accidents. This study reported the first data involving students enrolled from three universities on a voluntary basis for alcohol consumption evaluation. Alcohol consumption was assessed through questionnaires and EtG quantification in hair (hEtG) carried out by liquid chromatography-mass spectrometry (LC–MS/MS) analysis after a solid-phase extraction (SPE) purification step. The results of our study demonstrated that 77.1% of samples tested negative for hEtG or displayed hEtG ≤ 5 pg/mg. Particularly, the student population was not characterized by samples with hEtG indicative of chronic excessive consumption (hEtG ≥ 30 pg/mg). No significant association was identified between biological sex, among the degree course/the year attended, nor in relation to BMI or smoking/coffee consumption. Among the obtained results, it was worth noting that the comparison of self-reporting abstinence from tobacco and coffee accounted for 65.3% and 16.7%, respectively, while only 2.8% of the total declared abstinence from alcohol. The current study has uncovered a significant level of interest among students in this analysis and its interpretation. This suggests that implementing public health promotion activities within a university setting could be beneficial.

**Supplementary Information:**

The online version contains supplementary material available at 10.1007/s12024-023-00727-x.

## Introduction

In many cultures, ethanol is widely accepted, even with its psychotropic nature and potential for dependence. Alcohol consumption can be categorized into three groups: abstinence (no consumption), occasional consumption, and chronic excessive drinking (exceeding 60 g of ethanol per day) [1]. Worldwide alcohol harmful use accounts for 5.3% of total deaths per year, with a predominant involvement of adults aged 20–39 years old [2]. For this reason, in some clinical and forensic settings, it is fundamental to prove alcohol abstinence, e.g., liver transplantation, child custody proceedings, workplace and safety monitoring programs, and driving license renewal. Several indirect alcohol biomarkers have been used in the past to prove abstinence or repeated or excessive ethanol consumption. In the last years, a direct product of the non-oxidative metabolism of ethanol, ethyl glucuronide (EtG), has been used as a reliable biomarker to monitor abstinence or occasional use or confirm chronic ethanol consumption. EtG can be measured in blood, urine, and hair. EtG determination in hair (hEtG) has proven to be the most effective tool in expanding the detection window for alcohol excessive chronic consumption [3, 4]. The last revision of the Society of Hair Testing (SoHT) [5] consensus document defined three intervals for hEtG: EtG ≤ 5 pg/mg is considered not contradicting self-reported abstinence, a concentration of > 5 pg/mg strongly suggests repeated alcohol consumption, and a concentration of ≥ 30 pg/mg strongly suggests chronic excessive alcohol consumption. However, specific sub-populations might not reflect the consumption pattern of the general population and might require tailored cut-offs. Within this context, university students constitute an intriguing demographic. Young individuals often adjust their alcohol consumption rapidly in response to external factors such as participation in social events, increased autonomy during their studies, or cohabitation with others. Nonetheless, excessive alcohol consumption can have detrimental effects on academic performance, interpersonal relationships, and the risk and severity of accidents [6, 7]. Alcohol consumption among adolescents has been taken into consideration by several studies, and it is recognized as a serious issue across Europe. The European School Survey Project on Alcohol and Other Drugs (ESPAD) reported a high prevalence of alcohol consumption in young people, and particularly, heavy episodic drinking (five or more drinks on one occasion during the last 30 days) is estimated with a prevalence around 35% [8]. A recent systematic review assessing the pattern of alcohol consumption among undergraduate students in Ireland and the UK highlighted a high prevalence of hazardous alcohol consumption, drunkenness, and binge drinking [9]. In Italy, approximately 30% of the school students aged 15–19 years have reported binge drinking in the last month [10]. Some of these studies are based on surveys with self-reporting data, and under-reporting is frequently noticed [11]. To the best of the authors’ knowledge, only a few studies have been devoted to the objective assessment of ethanol consumption through EtG cut-off evaluation among the academic student population [12–14].

The aim of the present study was to assess alcohol use in a cohort of students from the geographical areas of Northern Italy through hEtG. This group represents a particular cluster that might reflect neither the consumption pattern of the general population nor of the at-risk population. As a secondary goal, we aimed at describing the habits of alcohol consumption also in relation to degree courses and attended year.

## Materials and method

### Design of the study

Hair samples were collected between 2021 and 2022 among students attending university courses in three Northern Italian regions. The study was submitted and approved by the Bioethics Committee of the University of Bologna (Prot. n. 76,007 of 26/03/2021) and has been performed in accordance with the ethical principles of the Declaration of Helsinki. Students from the Universities of Bologna, Parma (Emilia-Romagna region), Pavia (Lombardy region), and Turin (Piedmont region) were asked to participate. Inclusion criteria were the attendance of an academic degree course, a minimum age of 18 years, and the presence of head hair. Hair from other body districts was not considered. Exclusion criteria were relevant neurological, psychiatric, cardiovascular, pulmonary, endocrinological, or neoplastic diseases. Dyed and bleached hair and samples with a hair length lower than 3 cm were excluded from the study. Participants provided their informed consent before participating in the study. They were then asked to anonymously complete a general questionnaire, which included information on gender, height, weight, degree course, year of study, and their tobacco and coffee consumption habits. Ethanol consumption data was collected through self-reports (see Supplementary Information), where participants indicated the type of beverages typically consumed (such as wine, beer, plain spirits, cocktails, and aperitifs) and the frequency of consumption.

### Chemicals and instrumentation

Methanol, acetonitrile, and formic acid (all LC–MS grade) were purchased from Merck (Darmstadt, Germany), while ammonium acetate was obtained from Sigma-Aldrich (Saint Louis, MO). Strata-X-A solid-phase extraction (SPE) cartridges (60 mg, 3 ml) were acquired from Phenomenex (California, USA). Water was purified by PURELAB Chorus ELGA Veolia (High Wycombe, UK).

The reference materials, ethyl-β-D-glucuronide and ethyl-β-D-glucuronide-D_5_ (IS), were purchased from Sigma-Aldrich (Saint Louis, MO). Working solutions were prepared at a concentration of 100 ng/ml in methanol and stored at – 20 °C. Mobile phases consisted of 20 mM ammonium acetate at pH 6 in water (mobile phase A) and acetonitrile (mobile phase B). Flow rate was set at 0.3 ml/min.

Analyses were performed by LC–MS/MS (Xevo TQD, Waters, Milford, USA) via an electrospray ion source (ESI) operating in negative ion mode. A zwitterionic HILIC LC column (Poroshell 120, 2.1 × 100 mm, 2.7 μm), maintained at 30 °C was used for the separation. Gradient elution was as follows: 90% B for 1 min, 80% B from 1 to 7 min, held for 3 min, and equilibration for 5 min. Injection volume was 8 µl. The analytes of interest were detected in multiple reaction monitoring (MRM) mode, monitoring the following transitions: m/z 221 → 75, 221 → 85, and 221 → 113 and ETG-d5 (m/z 226 → 75 and 226 → 85). Cone voltage was 30 V for all, and collision energy was 18 V except for m/z 221 → 113 (collision energy: 12 V). Autosampler was kept at 10 °C. Data analysis was performed by MassLynx software (Waters, Milford, USA).

### Sample collection and preparation

Subjects were invited to participate in the study after a brief presentation of the project’s objectives and methodology, which was given prior to the attended lesson. Hair samples were obtained by cutting them as close to the scalp as possible from the vertex region of the head. These samples were then stored in paper envelopes at room temperature until analysis, for a maximum period of 2 months. The analysis focused on the proximal 3 cm of each hair sample. For calibration curves and validation, human hair samples were collected from children aged 3 and 4 years and prepared by creating a homogenized pool of hair. Samples and calibrators/controls were processed by adapting the procedure described in ref. [15]. A lock of hair was washed with 10 ml dichloromethane and 10 ml of methanol for 10 min each. Samples were left to dry at room temperature overnight and then cut into small pieces (1–2 mm) with scissors. For the extraction, 100 mg of hair was weighted, spiked with 30 µl of IS, and soaked in 1 ml of deionized water. Calibrators and QC were added to a proper amount of EtG. Samples were incubated overnight at room temperature, followed by ultrasound extraction for 2 h, at 50 °C. After centrifugation, 1 ml of the supernatant was submitted to solid-phase extraction (SPE). Cartridges were conditioned with 2 ml of methanol and 2 ml of deionized water. After sample loading, cartridges were rinsed with 1 ml of 5% NH_4_OH and 2 ml methanol. To remove all residual liquid, a strong vacuum was applied for 15 min. Elution was performed by 2 ml of 2% formic acid in methanol. The eluate was evaporated to dryness under a stream of nitrogen at 50 °C and then reconstituted in 150 μl of mobile phases A/B, (10: 90 v/v).

### Method validation

Selectivity, linearity, sensitivity, precision, accuracy, matrix effect, and stability were considered for validation. Selectivity was assessed by analyzing ten blank samples without IS and three blank samples with IS to check for interfering signals. Linearity was evaluated in the 5–45 pg/mg range of concentrations (5, 7, 10, 20, 30, 45 pg/mg). Four calibration batches were analyzed on 4 non-consecutive days. Accuracy, precision, matrix effect, and stability were calculated on quality controls (QC) prepared at three different concentrations (5, 15, and 25 pg/mg). QC were analyzed in two replicates for each concentration per day (intra-day precision) and on 4 non-consecutive days (inter-day precision). Precision was calculated as relative standard deviation (RSD) both intra-day and inter-day. Accuracy was calculated as percentage bias. The extraction procedure was tested on samples from proficiency tests (*n*.6) with authentic hair material. Limit of detection (LOD) and limit of quantification (LOQ) were determined by a signal-to-noise ratio of 3 and 10, respectively, and experimentally verified by spiking the calculated amount. Matrix effect was identified by comparing the ratio of peak areas of samples pre- and post-extraction and expressed as a percent. EtG stability was assessed in processed samples after 24 h at 10 °C by calculating the percent deviation on freshly prepared samples.

### Data analysis and statistics

We gathered data on age, biological sex, weight, height, degree program, academic year, smoking habits (cigarettes per day), daily coffee consumption, and alcohol-related behaviors through questionnaires. The degree programs were re-categorized into bachelor’s and master’s degrees. The data analysis involved classifying the collected information into predefined intervals. BMI was classified as BMI < 25 (normal) or BMI > 25 (overweight); smoking habits were classified as non-smokers, smokers of 1–9 cigarettes/day, and smokers over 10 cigarettes/day. Coffee drinking was classified into no consumption, up to 5 coffees/day (up to 400 mg caffeine) [16], and over 6 coffees/day. The self-reported frequency of ethanol consumption was pre-categorized into four groups based on the number of drinking occasions per week: no alcohol consumption, alcohol consumption on 1–2 days/week, alcohol consumption on 3–5 days/week, and alcohol consumption on all 7 days of the week. Hair EtG concentrations were classified according to the guidelines outlined in the Society of Hair Testing Consensus document. In addition, data were also grouped for negative samples (hEtG < LOD) and LOD < hEtG ≤ LOQ (5 pg/mg). No toxicological data exceeded the upper limit of 30 pg/mg. Congruence between self-reporting of alcohol consumption and hEtG levels was evaluated.

Normality and non-parametric statistics were assessed by Sktest (*p* > 0.05). Descriptive statistics was provided for all data, by the mean and standard deviation (SD) and/or by the median and interquartile range (IQ), for non-parametric variables. hEtG levels were compared among males and females and bachelor’s/master’s degrees by means of a non-parametric *t*-test (Mann–Whitney test). A similar comparison was performed on the basis of the reported frequency of ethanol consumption, by non-parametric ANOVA (Kruskal–Wallis test).

Statistical associations were attempted between hEtG data, divided into the 3 predefined concentration intervals (hEtG negative, hEtG ≤ 5 pg/mg, 5 < hEtG ≤ 30 pg/mg) and the following parameters, by means of chi-square analysis: biological sex, BMI category, degree course, year of course, smoking habits, coffee consumption, and frequency of ethanol consumption.

To evaluate participants’ awareness of their consumption patterns, we conducted a Spearman correlation analysis between hEtG levels and the self-reported frequency of ethanol consumption among questionnaire respondents. Furthermore, we calculated the congruence between the reported frequency of ethanol consumption and the interpretation of hEtG levels based on the Society of Hair Testing (SoHT) cut-off values.

For all analyses, a *p* < 0.05 was set for significance. Statistics was performed by Stata (StataCorp LP, version 14.0, Texas, USA) and images by Prism (GraphPad Software, LLC, version 9.3.0).

## Results

### Method development and validation

The analytical method described in ref. [17] was applied after the assessment of validation parameters. EtG and EtG-d5 eluted with a satisfactory retention time, 6.81 and 6.78 min respectively. No interfering peaks were detected. The method was successfully validated for all the analyzed parameters. Extraction efficiency calculated on proficiency test samples proved adequate and within the limits. Limit of detection (LOD) and limit of quantification (LOQ) were 3 pg/mg and 5 pg/mg, respectively. Linearity was from 5 to 45 pg/mg, the weighing factor was 1/*x*, and *R*^2^ is always > 0.99. Accuracy, precision, sensitivity, RSD of the angular coefficient and of the R^2^, stability, and matrix effect are detailed in Table 1 of Supplementary Information.

**Table 1:** Characteristics of the respondents to the questionnaires (age, weight, height, BMI)

	**Mean (SD)**	**Median (IQ)**	**Parametric**
Age (years)	23 (3.7)	22 (24.0–21.0)	No
Weight (kg)	63.5 kg (10.6)	62.0 (7.01–55.5)	Yes
Height (cm)	169.6 cm (9.1)	168.5 (177.0–164.0)	Yes
BMI	22.0 (2.8)	22.0 (23.5–20.0)	No

*BMI* body mass index, *SD* standard deviation, *IQ* interquartile range

### Application on a student population

A total of 114 hair samples were collected from students during the lessons or immediately after; 9 samples were excluded from the analysis due to insufficient material.

#### Students’ characteristics

The study covered the 98% of representativity of the student population that was asked to join the research. Students were enrolled mostly from scientific and medical university courses (20.9% biology, 22.9% medicine and surgery, 8.6% midwifery, 6.7% laboratory technician, 5.7% dentistry), while other course degrees accounted for a total of 28.5% of the participants. Samples were represented mostly by females (69.4%), with a median age of 22 years (IQ: 21–24), being 19 the minimum and 42 the maximum age. BMI values ranged from 16.8 to 34.9. Mean and median values of age, weight, height, BMI, and descriptive characteristics of the student population are reported in Tables 1 and 2. The study carrier was covered uniformly. The year of study varied from the first (8.6%) up to the sixth (11.4%) in the case of medicine and surgery course. Students attending the second year of study were 17.1%, 24.4% attended the third year, and 25.7% and 12.9% attended the fourth and fifth, respectively. Two subjects reported abstinence from alcohol (2.8%), while 61.1% of the respondents indicated alcohol consumption on 1–2 days/week (for more details, refer to Table 2). In total, repeated alcohol consumption, whether limited to 1–2 days or up to 5 days/week, accounted for 94.4% of the subjects. Only two subjects reported consuming ethanol every day of the week. Regarding the type of alcoholic beverages consumed, 35 subjects reported aperitifs, 47 mentioned beer, 29 mentioned cocktails, 35 mentioned spirits, and 55 mentioned wine. Furthermore, 87.5% of students reported consuming more than one type of alcoholic beverage, with the most common combination being beer, wine, and spirits.

**Table 2:** Characteristics of the respondents to the questionnaires (biological sex, BMI, smoking habit, coffee consumption, ethanol consumption)

	**Descriptive results**	**Respondents (72 in total)**
Biological sex	Females	50 (69.4%)
	Males	22 (30.6%)
BMI	≤ 25	66 (91.7%)
	> 25	6 (8.3%)
Smoking habit	Non-smokers	47 (65.3%)
	Smokers 1–9 c/day	22 (30.6%)
	Smokers ≥ 10	2 (2.8%)
	Electronic	1 (1.3%)
Coffee consumption	No coffee	12 (16.7%)
	1–5 coffees/day	60 (83.3%)
	> 6 coffees/day	–
Ethanol consumption	Abstinence	2 (2.8%)
	1–2 days/week	44 (61.1%)
	3–5 days/week	24 (33.3%)
	7 days/week	2 (2.8%)

*BMI* body mass index, *c/day* cigarettes/day

#### EtG concentrations in hair

**Fig. 1:**
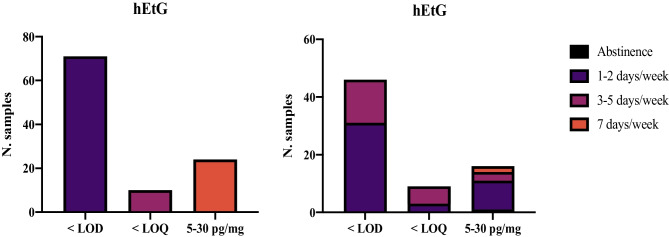
hEtG results in the student population, classified into three categories (left), and in relation to the declared frequency of ethanol consumption (right). LOQ: limit of quantification (5 pg/mg). LOD: limit of quantification (3 pg/mg)

**Fig. 2:**
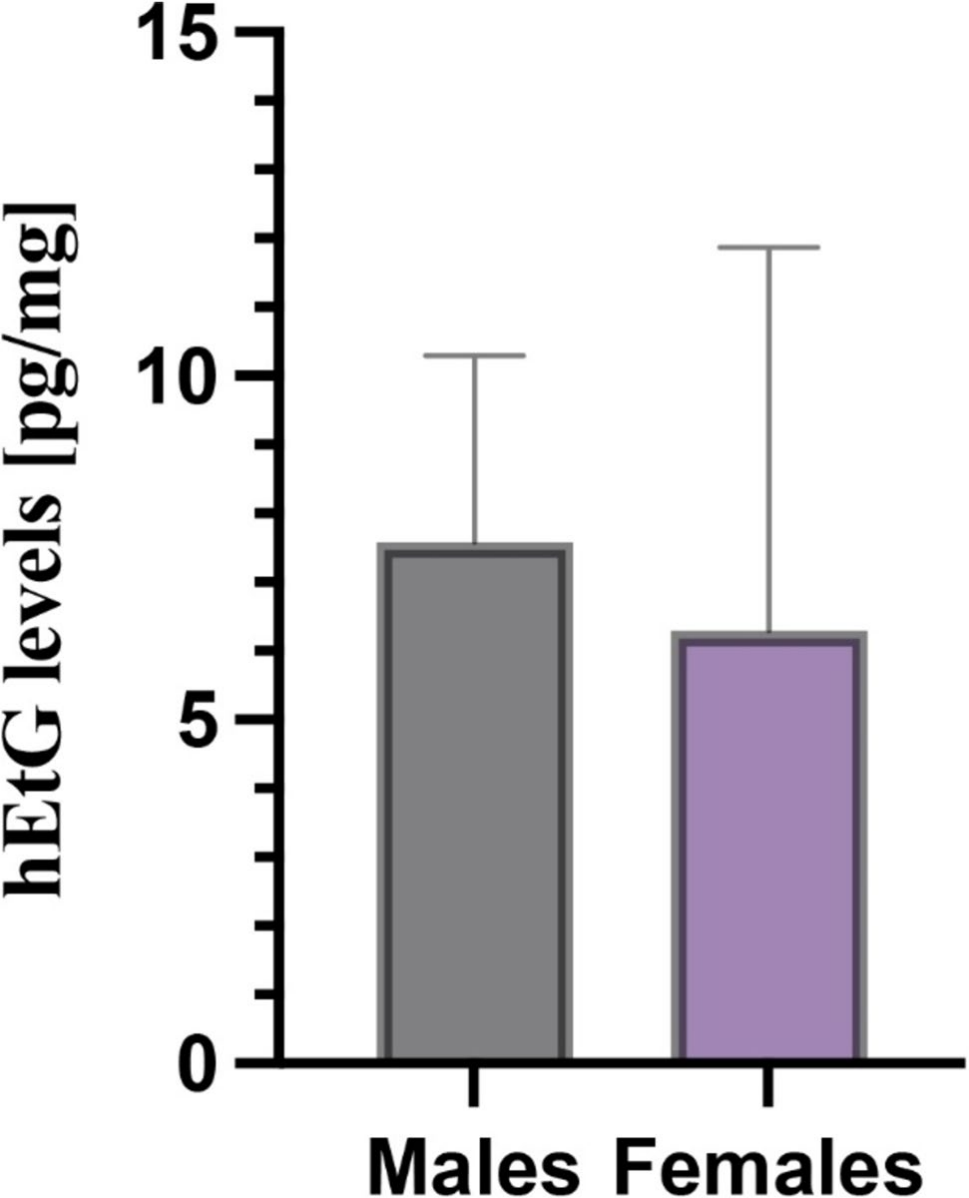
hEtG levels in the male and female population

Ethylglucuronide was analyzed in 105 hair samples, 71 of which (67.6%) displayed negative hEtG levels (< LOD), 10 (9.5%) displayed LOD ≤ hEtG < LOQ (5 pg/mg), and 24 (22.8%) presented 5 ≤ hEtG < 30 pg/mg. (Fig. 1). Mean and median hEtG were 8.3 pg/mg (SD: 4.3) and 6.4 pg/mg (IQ: 9.7–5.8), respectively (data was non-parametric). Highest hEtG concentration was 22.6 pg/mg. hEtG gender distribution was also calculated. In males, mean hEtG level was 7.9 pg/mg (SD: 2.4), with a median of 7.6 pg/mg (IQ: 10.3–5.8), while in females, mean and median levels were 8.9 pg/mg (SD: 5.5) and 6.3 pg/mg (IQ: 11.9–5.4), respectively. The Mann–Whitney test showed no statistically significant difference (Fig. 2). Mean hEtG level was 8.8 pg/mg (SD: 1.4) among bachelor’s degree students and 7.7 pg/mg (SD: 1.0) among master’s degree studies, with no statistically significant difference between the groups.

By comparing toxicological data deriving from males and females and hEtG intervals, no significant association was identified (*p* = 0.857). Also, no association was found between hEtG category and the degree course (*p* = 0.425), bachelor’s/master’s degrees (*p* = 0.466), the year attended (*p* = 0.478), the category of BMI (*p* = 0.912), the smoking habit (*p* = 0.287), nor the coffee consumption (*p* = 0.469). As expected, the category of hEtG was significantly associated with the reported frequency of ethanol consumption (*p* = 0.016) (Table 3). Spearman’s correlation between the frequency of self-reported ethanol consumption and the hEtG levels, performed only on samples with hEtG > 5 pg/mg, showed a statistically significant association *p* = 0.033, with a rho of 0.538. The calculated media and SD for each category of reported alcohol consumption were 7.6 pg/mg (SD 3.7), 14.1 pg/mg (SD 7.5), and 8.4 pg/mg (SD 1.2), for 1–2 days/week (occasional drinkers), 3–5 days/week (repeated drinkers), and 7 days/week respectively. Median values were 6.3 pg/mg (IQ: 8.6–5.3) in subjects reporting occasional drinking and 11.3 pg/mg (IQ: 22.6–8.4) in subjects declaring repeated drinking. For self-declared abstinents (*n*.2), one sample was negative, and one sample displayed an hEtG concentration of 5.8 pg/mg. Congruence of the self-reported frequency of ethanol consumption with the hEtG result interpretation according to the SoHT cut-offs is reported in Table 4.

**Table 3:** The table summarizes the hEtG concentration intervals in relation to the self-reported ethanol consumption

hEtG concentration	Abstinence	1–2 days/week	3–5 days/week	7 days/week
≤ LOD (3 pg/mg)	1 (50%)	31 (70.5%)	15 (62.5%)	0
≤ LOQ (5 pg/mg)	0	3 (6.8%)	6 (25%)	0
5–30 pg/mg)	1* (50%)	10 (22.7%)	3 (12.5%)	2 (100%)
Total	100%	100%	100%	100%

^*^One subject self-declaring abstinence was found with an hEtG concentration of 5.8 pg/mg

**Table 4:** Congruence between self-reported frequency of ethanol consumption and hEtG result interpretation according to the SoHT cut-offs

Self-reported consumption	EtG interpretation	Cases	% of confirmable cases
Abstinence or occasional drinking (1–2 days/week)	Confirmation of abstinence or rare occasional drinking (< 5 pg/mg)	35	77.8%
	Moderate drinking (5–30 pg/mg)	11	
	Chronic excessive drinking (> 30 mg/pg)	0	
Moderate drinking (3–5 days/week)	Abstinence or rare occasional drinking (≤ 5 pg/mg)	21	12.5%
	Confirmation of moderate drinking (5–30 pg/mg)	3	
	Chronic excessive drinking (> 30 mg/pg)	0	
Excessive drinking (7 days/week)	Abstinence or rare occasional drinking (≤ 5 pg/mg)	0	0%
	Moderate drinking (5–30 pg/mg)	2	
	Confirmation of chronic excessive drinking (> 30 mg/pg)	0	

## Discussion

Our study encompassed 105 university students collected from three distinct geographical regions in Northern Italy. These students were enrolled in various university courses, spanning the entire academic journey from the first to the sixth year. We employed biological sex as a statistical parameter for classification, considering the different metabolisms of alcohol. However, we recognize that gender patterns can indirectly influence alcohol consumption. Notably, our sample was predominantly composed of women, which may be attributed to the specific courses selected. For instance, courses such as medicine and surgery, biology, and midwifery consistently exhibit a pronounced prevalence of female students, as indicated by statistical data [18]. This prevalence may, at least in part, account for the high percentage (67.6%) of samples that tested negative (< LOD) for hEtG. Previous studies have already estimated lower alcohol consumption among the female population [12, 13, 19]. Among the samples that tested positive for hEtG, females exhibited a lower mean concentration, offset by greater variability in the results. Consequently, no statistically significant difference could be observed between the genders. This data was also confirmed by the results regarding the Italian population of school students [11]. Taken into consideration the parameters such as type of university course, year of carrier, tobacco, and coffee consumption, our study confirms that no association was observed with alcohol consumption [13, 20]. Regarding the impact of BMI, previous studies have reported significant differences in hEtG levels based on this index [21]. The relationship between alcohol consumption and BMI is multifaceted. In fact, while moderate alcohol consumption may not lead to significant weight gain in some individuals, heavy and excessive drinking can contribute to obesity and other health problems. However, our study did not confirm these findings, possibly due to the limited number of subjects in the obesity groups (grade 1–2-3).

The indicator that young people perceived alcohol consumption as posing little or limited risk to their health emerged through a comparison of self-reported data on tobacco, coffee, and alcohol habits.

Alcohol and tobacco are often consumed together to reinforce each other’s effects. However, co-use of alcohol and tobacco is associated with significantly higher health risks. It can increase the risk of oral, throat, esophagus, and lung cancers, cardiovascular diseases, and respiratory problems. Coffee is commonly consumed for its stimulant properties, and the co-consumption with alcohol may mask some of the sedative effects of alcohol, leading to risky behaviors, such as drinking and driving, because of the underestimation of the level of impairment.

In our study population, only 2.8% reported abstaining from alcohol, while 65.3% and 16.7% reported tobacco and coffee abstinence, respectively. Besides, the ESPAD data [22] on the perceived availability of retrieving substances among European young people reported a 78% of easiness for alcohol, compared to 60% of cigarettes and 32% of cannabis. Italian data were in line with European statistics, with a perceived availability of 83% from “fairly easy” to “very easy” in retrieving alcohol. In our cohort, self-reported alcohol consumption was generally moderate, with 94% of participants indicating alcohol consumption on 1–2 days/week (61.1%) and 3–5 days/week (33.3%). Only two subjects reported consuming alcohol daily. This pattern is supported by our analytical findings, which yielded a median hEtG concentration of 6.4 pg/mg, with values ranging from 5.3 to 22.6 pg/mg. As previously mentioned, there is limited literature available on the objective assessment of alcohol consumption among university students, which hinders a comprehensive evaluation of our findings. Nevertheless, the published data align with our study, substantiating the absence of widespread chronic excessive alcohol consumption within student populations. Actually, Oppolzer et al. tested students from 9 Portuguese universities and reported hEtG median values between 8.18 and 21.13 pg/mg [12]. The main difference compared to other studies relies on the hEtG maximum concentration, which reached up to 153 pg/mg [13] and 180 pg/mg [14] in the analyzed student populations. The reasons for this discrepancy may stem from variations in alcohol consumption habits across different geographical regions, as partially evidenced by the differences in median values observed in Portugal. Additionally, the sample size available for our study could have played a role. As a future perspective of our research, it would be highly desirable to enhance the study by increasing the sample size and expanding the regional selection. A selection bias must be taken also into consideration, due to moderate drinkers being more inclined than heavy drinkers to participate, since our study did not include any benefit (i.e., economical compensation). In fact, a discrepancy has been observed between quantitative results of self-declared heavy drinkers (7 days/week) and hEtG of self-declared repeated drinkers (3–5 days/week), with a mean hEtG of 8.4 pg/mg and 14.1 pg/mg, respectively. This might confirm that alcohol consumption is very often underestimated and that the inclusion in our study of reports from collateral informants [23], besides the primary research subjects, could help in the research. In our study, although the self-reported frequency of ethanol intake and the hEtG ordinal category appeared to be associated based on chi-square analysis, a positive Spearman correlation was found within quantifiable samples. This suggests a level of awareness among participants regarding their consumption habits. This seemingly contradictory result can be explained by an overall alignment between self-reported intake and objective hEtG measurement. However, this alignment may have limited awareness, particularly when focusing on repeated or heavy drinkers, as previously observed in adolescent studies [11]. Moreover, an under-reporting of heavy drinking and a rather low proportion of positive agreement between reported drinking and hair analysis (around 50%) were also documented by Fendrich and colleagues [23].

When evaluating the alignment between self-reported consumption and hEtG categorization using the established cut-offs, the majority of subjects who reported abstinence or occasional ethanol consumption (1–2 days/week) tested negative or had hEtG levels < 5 pg/mg. This strongly confirms the accuracy of these levels in diagnosing ethanol abstinence and infrequent occasional drinking. However, when examining those who reported repeated and excessive drinking, most students misjudged their habits as occasional or moderate, respectively. Confirmation was achieved in only 12.5% and 0% of cases. In fact, a consumption of alcohol of 7 days/week was declared only by two students, which is in contrast with the results from the observed quantitative hEtG measurements. An important issue in the evaluation of data relies on cut-off use and interpretation. The adopted cut-off of 5 pg/mg (“not in contradiction to abstinence”) does not mean that “abstinence can be proven.” There is a difference between “interpretation” and “scientific facts”; thus, the 5 pg/mg cut-off is a convention and is used for interpretation in specific application fields, e.g., for the purpose of controlling abstinence for renewal of driver’s license after excessive consumption and drunk driving. When the cut-offs are used in contexts other than they were initially proposed, they should be carefully interpreted. In our student population, low amounts of alcohol were consumed (number of “standard drinks per week”), but this consumption did not result in positive EtG concentrations in hair. Thus, consumption could not be totally excluded, if no EtG was detectable. Probably, analysis of EtG in hair, when applied to a low-risk population, should be performed at the highest method sensitivity, in order to detect alcohol consumption at such low levels of 2–5 drinks per week.

To evaluate alcohol consumption among young people raises several ethical considerations; young people often face social pressure to drink alcohol, and this raises questions about peer influence and the role of parents, educators, society, and even advertising/marketing in promoting responsible drinking or abstaining. Also, the knowledge of the long-term consequences of alcohol abuse such as addiction, liver damage, impaired brain development, and accidents related to intoxication is a critical issue considering their decision-making abilities that may not be fully developed.

In our current study, we did not standardize alcohol consumption based on alcoholic units due to the challenges in calculating alcohol content in aperitifs and cocktails, which are the most consumed drinks among young Italians (56% in our sample). While this limitation may restrict the ability to make general comparisons of results, the data on alcohol consumption patterns throughout the week can still provide valuable insights into identifying high-risk behaviors among young people. Frequency of hair washing and use of cosmetic treatment was not analyzed in our study, and this might also have influenced the hEtG concentration, though all dyed and bleached hair samples were excluded. The sampling time did not take into consideration any academic examination which might have been connected to higher or lower ethanol intake nor the post-pandemic era influence. Finally, binge drinking, which represents the consumption of large amounts of alcohol on the same occasion, is very common in young adults [24, 25]. However, analysis of EtG in hair is able to detect only a relatively high amount of daily ethanol intake making the evaluation of occasional and/or binge drinkers very difficult. For this reason, the evaluation of alcohol consumption among young students may need the integration of data from different biological markers or matrices [26].

The present study also highlighted the strong interest of students in this analysis and its interpretation. This underscores the importance of developing targeted information campaigns for university students regarding alcohol-related risks. Such campaigns can play a crucial role in the early detection and intervention of high-risk alcohol consumption behaviors. Preventing alcohol consumption among young people is a multifaceted endeavor that requires the collaboration of parents, schools, communities, and policymakers. By implementing a comprehensive approach that combines education, enforcement, and support, it is possible to reduce the rates of alcohol consumption and related harm among young individuals. Also, the early intervention through counseling and treatment for young people who may be at risk of developing alcohol-related problems would be advisable in an educational setting.

## Conclusions

The objective determination of hEtG associated to self-reported alcohol consumption provided useful data on the drinking behavior of young adults. Considering that alcohol consumption among this very sensitive population has deleterious consequences on health and the observed general support to the study with high levels of interests, the university setting may represent the ideal frame for alcohol prevention strategies. Based on the fact that this study emphasized a very low level of perceived risk for alcohol consumption, prevention strategies should include both program development of appropriate information about alcohol, including information on the short-term effects and long-term consequences of its use, and the development of personal, social, and resistance skills to help students identify internal/external pressures (e.g., anxiety and stress, peer pressure, and advertising) and to give students the skills to resist these pressures. Finally, also, policy strategies may play a role in reducing the commercial and social availability of alcohol for young people.

## Key points

1. Consumption of alcohol was reported by 94.4% of the subjects.
2. Declared abstinence from alcohol was lower than tobacco and coffee abstinence.
3. Students displayed hEtG ≤ 5 pg/mg in 77% of cases.
4. No association was identified among the degree course or the attended year.

## Supplementary Information

Below is the link to the electronic supplementary material.

Supplementary file1 (DOCX 275 KB)

## Data Availability

N/A.
