# Supplementary material for: Determination of ethyl glucuronide in hair and self-reported alcohol consumption in university students
Source: Forensic Sci Med Pathol. 2023 Oct 5;20(3):769–77. doi: 10.1007/s12024-023-00727-x (PMC11525380; doi:10.1007/s12024-023-00727-x)

**Figure 1**.


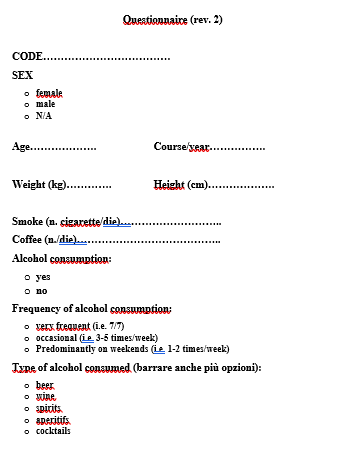
Questionnaire proposed to volunteers before sample collection.

| **Analyte** | **LOD**  **(pg/mg)** | **LOQ**  **(pg/mg)** | **Linearity**  **(pg/mg)** | **Angular coefficient**  **RSD, %**  **n.4** | **R^2^**  **RSD, %**  **n.4** | **Stability, %**  **24 h, 10°C** |
| --- | --- | --- | --- | --- | --- | --- |
| EtG | 3 | 5 | 5- 45 | 8 | 0.4 | 115 |
| **QC level**  **(pg/mg)** | **Precision**  **RSD, %** | | **Accuracy Bias, %** | **Matrix effect, %** |  |  |
|  | **Intraday** | **Interday** |  |  |  |  |
| ***Low: 5*** | 16 | 12 | -3 | 104 |  |  |
| ***Intermediate: 15*** | 3 | 6 | 6 | 107 |  |  |
| ***High: 25*** | 4 | 12 | 9 | 112 |  |  |

**Table 1**.

Summary of the results of the validation study. LOD: limit of detection. LOQ: limit of quantification. RSD: relative standard deviation. QC: quality control. Stability reported on QC as average value.

**Figure 2**.

Chromatograms of hEtG LC-MS/MS analysis. A: blank hair spiked at 20 pg/mg; B: blank hair spiked at 5 pg/mg (QC low); C: calibration point spiked at 30 pg/mg; D: real negative sample; E: hEtG positive sample at the concentration 15.7 pg/mg. In F, an example of a calibration curve.


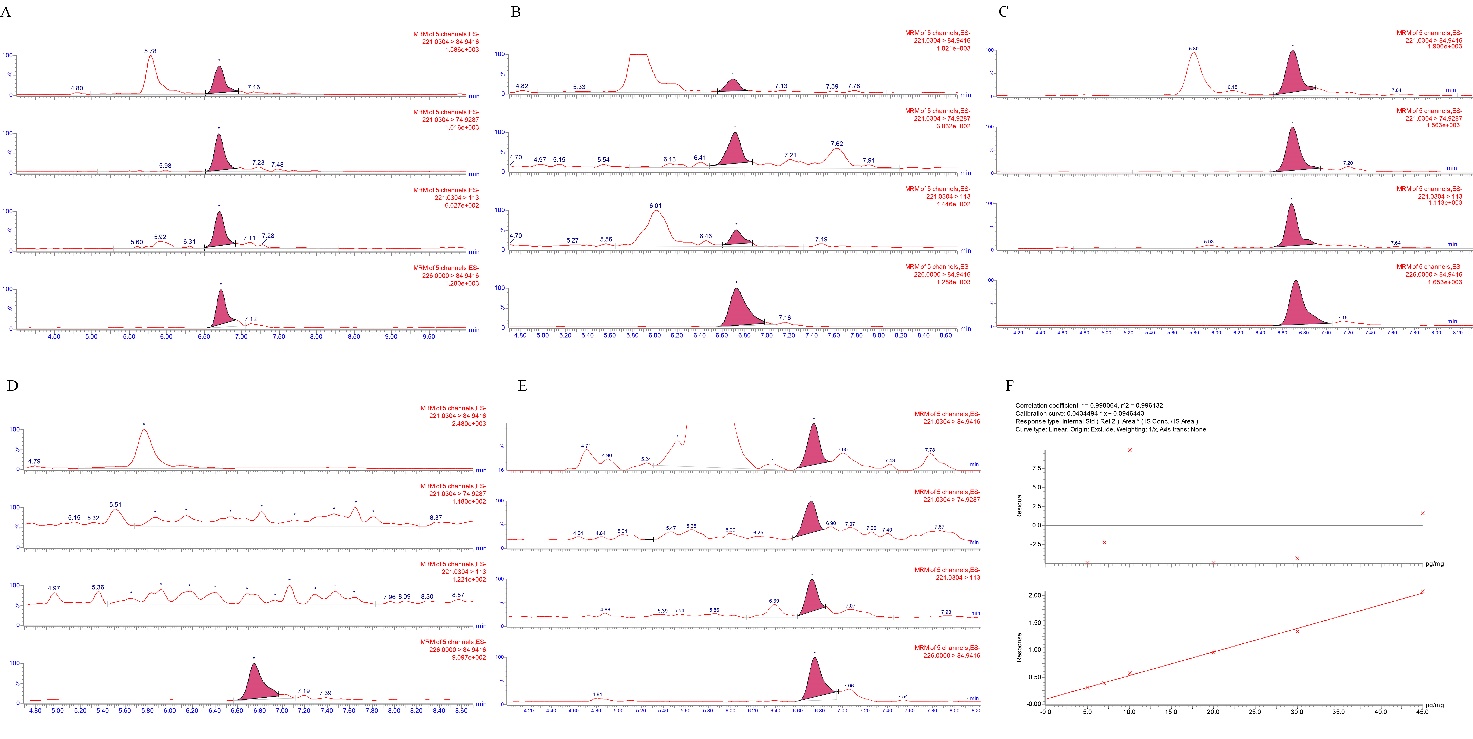

Supplement: Supplementary file 1 — Supplementary file1 (DOCX 275 KB) [file 12024_2023_727_MOESM1_ESM.docx]
